# Migration, Stress and the Challenges of Accessing Food: An Exploratory Study of the Experience of Recent Afghan Women Refugees in Adelaide, Australia

**DOI:** 10.3390/ijerph17041379

**Published:** 2020-02-21

**Authors:** Foorough Kavian, Kaye Mehta, Eileen Willis, Lillian Mwanri, Paul Ward, Sue Booth

**Affiliations:** 1 College of Nursing and Health Sciences, Flinders University, GPO Box, Adelaide 2100, Australia; Foorough.Kavian@flinders.edu.au (F.K.); Kaye.Mehta@flinders.edu.au (K.M.); Eileen.Willis@flinders.edu.au (E.W.); 2 College of Medicine & Public Health, Flinders University, GPO Box, Adelaide 2100, Australia; Lillian.Mwanri@flinders.edu.au (L.M.); Paul.Ward@flinders.edu.au (P.W.)

**Keywords:** refugees, Afghani women, migration, food access

## Abstract

This study explored the migration and food experiences of Afghani women refugees residing in Adelaide, South Australia for 2 years or less. In-depth semi-structured qualitative interviews were conducted with 10 women between May and September 2017. The data were thematically analysed, and the Social Determinants of Health Framework was used to discuss the findings. Five key themes emerged from the data. In the transition country (Iran/Pakistan), respondents experienced (i) trauma, discrimination and exclusion and (ii) familiar food culture, but food stress. In the destination country (Adelaide, Australia) respondents experienced (iii) a sense of precariousness, (iv) unfamiliar food culture and (v) challenges in accessing halal food. Afghani refugees experienced considerable stressors both in the transition and the final destination country but for different reasons. In the transition country, stresses related to the lack of social services and support, discrimination, racism and poverty seemed to have affected their ability to afford food. In Australia stressors pertaining to socioeconomic, housing and employment precariousness, as well as difficulties in accessing halal foods were identified as challenges. Furthermore, food stress in Australia was associated with the cultural appropriateness of food, the complexity of the food system, and the women’s lack of skills and experiences in navigating the food system. With increasing refugee and immigration flows globally, it is necessary to acknowledge how food and social determinants intersect for refugee immigrants to ensure positive health outcomes.

## 1. Introduction

According to the United Nations High Commission for Refugees (UNHCR) the number of people worldwide who have been forcibly displaced from their homes has reached 71.44 million. This is an increase of 2.9 million since 2016 and is the result of civil war, invasion, persecution and violence [1]. A refugee is a person who is outside their own country and is unable or unwilling to return due to a well-founded fear of being persecuted because of their race, religion, nationality, membership of a particular social group or political opinion [2].

Australia receives more than 12,000 entrants yearly as refugees from conflict zones around the world through its humanitarian program with currently just over 92,000 people classified as either refugees or asylum seekers [3]. During the past decade there has been a sharp increase in the number of Afghani people migrating to Australia, so that they are now one of the fastest growing refugee groups in the country. According to the 2016 Australian Bureau of Statistics (ABS) census, there are 53,082 Afghani refugees or people claiming Afghan ancestry living in Australia, an increase from just under 28,597 recorded in the 2011 ABS Census [4]. The 2016 Census indicates around 12% of humanitarian visas were assigned to people from Afghanistan within the previous census time frame [5].

Factors underpinning the level of Afghani migration to Australia include the on-going civil war with the Taliban as well as drought [6,7]. While the majority have settled in Victoria, in 2018, South Australia was home to 5860 people of Afghani ancestry, the majority of whom were refugees [8]. Many of these refugees were subjected to trauma, including prolonged periods of deprivation, the loss of loved ones in violent circumstances or a hazardous escape from their homeland to their first destination of refuge. Afghani refugees to Australia, typically migrate via countries such as Iran and Pakistan, and may well have come via boat. The amount of time spent in these transition countries varies and can be as long as several years, while awaiting processing or illegal transfer to Australia. For example, in 2013–2014 Afghani refugees designated as Irregular Maritime Arrivals were in the top three countries applying for refugee status.

Once in Australia, Afghani refugees are often economically vulnerable as a result of their visa status. These refugees are on a temporary as opposed to a full humanitarian permanent protection visa and are denied access to the full range of social security service [9]. They receive a reduced social security payment set at 89% of the rate for Australian born unemployed citizens and they may also find it difficult to gain employment due to language difficulties [9].

The annual income for Afghani refugees on humanitarian visas in 2014 was $27,000, while the average Australian-born income at that time was $46,000 [10]. Refugees constitute the most economically disadvantaged migrant group and are a socially excluded segment of the Australian population, suffering poor health, including limited access to and utilisation of healthcare [11].

Migration also impacts on dietary patterns, eating habits and health [12]. Food habits play a central role in cultural identity and are stable and enduring [13]. After migration to a new culture, changes to food habits may occur and are termed dietary acculturation. Dietary acculturation defined as “when members of a migrating group adopt the eating patterns/food choices of their new environment” [14] is not a linear process but complex and dynamic [15]. Aspects of dietary acculturation can include food neophobia, that is the rejection of foods that are unknown [16] as well as negative health effects over time as a result of increased consumption of energy dense foods [17,18].

Refugees arriving in Australia encounter a new living and food environment that differs considerably from their home country and what they experienced in the transition country. The impact of migration on food amongst refugees, manifests in a variety of ways depending on the time elapsed since the arrival in Australia. There are many factors which influence refugees’ ability to establish healthy eating patterns such as low income due to unemployment, limited English language proficiency, lack of familiar food, transport difficulties, shopping practices and cooking methods [19].

The health of refugees and asylum seekers is known to be poorer than that of host populations, however, most public health studies have focused on mental and maternal and reproductive health [20]. Refugees and asylum seekers also show higher rates of physical health problems from infectious diseases and chronic non-communicable diseases [11]. This places them in a position of health inequity early in their resettlement. Social determinants such as unemployment, unsatisfactory accommodation and inability to speak the host country language well, add to this health inequity [21].

There is scant Australian literature on the food experiences of Afghani refugees and several studies have identified food insecurity in recently resettled refugees. (from Africa, Asia, Middle East, Europe and the former Yugoslavia) in Sydney, Melbourne and Perth [19,22,23]. Other studies have examined eating habits and dietary acculturation more generally among migrants to Australia, identifying both the coexistence of traditional diets along with a shift in dietary patterns to incorporate some processed foods such as soft drinks, crisps, and fast foods into their diets [15,17]. Some dietary acculturation studies focus on food habits after many years [12,24], with other studies focusing on food habits in the early phases (≈2 years) after migration [15,19,22]. Early phase dietary acculturation is important because specific challenges may exist that are not evident in latter phases such as lack of available familiar food and maintaining attachment to traditional food [15,19]. Focusing on the early stages of dietary acculturation may highlight important aspects of how new food habits develop in the adjustment journey for Afghan migrants to Australia.

To the authors’ knowledge, there are no Australian studies examining the food experiences of Afghani refugees in both the transition and the final destination countries. The purpose of this paper is to explore the migration journey and food experiences of Afghani women refugees (now resident in Adelaide) in both their transition and final destination countries. It is well known that the social environment in which people live has a profound effect on their health and wellbeing and this in turn is shaped by the distribution of power, money and other resources in society [25]. In the context of the migration experience specifically, social determinants such as income, education, conditions of employment and social exclusion act together in a powerful way to impact negatively on the health of individuals and communities [21,26]. The International Organisation for Migration argues that migration per se, can be considered a determinant of health in both the transition and final destination countries [27]. The concept of the Social Determinants of Health (SDOH) was chosen as a lens to help understand the various social determinants which affect Afghani migrants and their access to food because it embraces a broad view of health including the underlying and interconnected influences.

## 2. Study Design

A qualitative study employing face-to-face in-depth semi-structured interviews was conducted with 10 female Afghani refugees in Adelaide. Several assumptions underpin qualitative research including that the human world is created through interactions and therefore interpretive research seeks to understand deeply these interactions [28]; meanings and understandings attached to social interactions and phenomena are important because they provide rationales and justification for behaviour [29,30]; and truth is the lived experience of reality and is always subjective [29,30]. Young women were selected for interviews since they tend to take on the primary role in the family in terms of food acquisition and preparation. The inclusion criteria were Afghani female refugees who lived in Australia for 2 years or less. The target of 2 years immigration had been used in previous research to signify relatively new immigrant status [31]. It can be assumed that relatively new immigrants would experience considerable acculturation stress [32]. This will also ensure participants could accurately recall their food and life experiences in the first destination and the changes that followed. This study related to a larger international quantitative study investigating the experiences of food insecurity amongst Afghani refugees in Saskatchewan (Canada), Bern (Switzerland) and Adelaide (Australia) [33].

### 2.1. Recruitment

Recruitment employed snowball sampling and involved varied strategies with numerous organisations who work with refugees. A number of consultations with community leaders were undertaken, through the Middle Eastern Communities Council of South Australia, in order for them to understand the benefit of the study for their members and ultimately to endorse it. Once endorsed, several locally based organizations were approached, including educational and multicultural organizations working with young refugee people, and adult English learning institutions in a locality with a relatively high refugee population. Use of such adapted snowball sampling techniques as well as culturally sensitive approaches is effective and efficient in recruiting vulnerable populations [34]. After obtaining approval from these organisations, project information sessions were conducted in-house. The organisations then distributed flyers for relevant staff and clients. Interested participants then contacted the principal investigator (F.K.) and a study information sheet was provided. Consenting participants were interviewed at a convenient time and place such as a private space in the local library.

### 2.2. Data Collection

Research in English-speaking countries involving non-English speaking communities is increasing [35] and this is the case in Australia as researchers engage participants from multicultural and emerging communities. Interviews were conducted from May to September 2017 in the participants’ native language, Dari, by the principal investigator (F.K.) who is bilingual in Farsi/Dari and English. Whilst participants were offered the interview in either English or Dari, they all opted for Dari since they claimed that their English was poor. Using a bilingual interviewer who speaks both English and Dari produced more accurate data from participants who could talk about their experiences in their own language. Additionally, the bilingual interviewer could interpret meanings of some words in Dari that could not be translated exactly into English. The literature on the quality of translation in research suggests that a ‘good’ translation is one that does its job while inscribing in the receiving language the most relevant equivalent meaning from an original [36]. Further, translation is not a literal word-to-word process, rather, ‘it suffices to transmit the idea, the figure, the force’ [36]. Interviews were transcribed and translated into English by the bilingual researcher, and thus external validation of the transcriptions was not undertaken.

To obtain a comprehensive understanding of views, opinions, experiences and practices of Afghani refugee families about the challenges of availability, accessibility and sustainability of accessing nutritious and culturally appropriate food, a set of open-ended questions formed the basis of the interviews about the social determinants of health in both the transition country and Australia. Questions were adapted from the original study designed by our collaborators in Canada [33]. The interviews involved exploring conditions that can expose migrants to increasingly negative health outcomes including access to health care, education and safe working and living conditions. This also involved discussions of circumstances surrounding their legal, social, cultural and communication challenges as well as the settlement experiences in their transition country and in Australia, including sociocultural, socioeconomic and environmental factors which impact (positively and negatively) on their perceptions of their food insecurity. Interviews focused on the impact of their previous experiences in the transition country of residence and their integration into Australian society, as well as similarities and differences in availability, accessibility and usage of culturally acceptable food. In qualitative research, the concept of interview ‘data saturation’ is used to help determine the number of interviews conducted. Data saturation is defined as the point at which ‘no new information or themes are observed in the data’ [37]. In this study, interviewing continued until the researchers deemed data saturation had been reached at 10 interviews.

### 2.3. Ethics Approval

This research was approved by the Flinders University Social and Behavioural Research Ethics Committee, Approval Number 7251.

### 2.4. Data Analysis

All interviews were audio-recorded to accurately capture the participant responses. The principal investigator (F.K.) also kept field notes in order to capture the non-verbal communication and to get an immediate ‘sense’ of the data emerging from each interview. She first listened to audiotaped interviews in Dari in order to gain a sense of participants’ core meaning and understandings. Summaries of each interview were presented to the rest of the research team. Translated quotes were also presented from the interviews as part of these summaries, or vignettes. These vignettes were then discussed in team meetings in order to develop an initial coding frame, and to get a sense of the key themes in the data. Once the research team had a good sense of the data, based on summaries of the audio recordings in Dari, all interviews were then fully translated into English and transcribed (by F.K.).

We undertook two consequential layers of analysis: open coding and focused coding [38,39]. Data were primarily coded by F.K., but also cross-coded by K.M. and S.B. to increase analytical rigour. Our open coding provided a description of the issues or themes arising from the data, from the perspective of the participants. This was undertaken throughout the data collection process, and our initial open coding informed the content of subsequent interviews. Each interview was transcribed directly (after translation) after the interview so that the data analysis and collection could be compared. The process of open coding followed the recognised stages: breaking down, examining, comparing, conceptualising and categorising data [40]. When open coding, words or sections of text were coded using the actual content used by participants (NVivo codes), or by grouping similar words conceptually.

Focused coding was undertaken by grouping the open codes into larger categories, which is more directed and selective than open coding, since it explains larger bodies of text by using significant and/or frequent codes [41]. This process involved an iterative process of slotting each of the initial open codes into larger categories, based on their ‘semantic fit’ or the ways in which they seemed to be related to a similar idea or issue. These initial focused codes were large and needed to be reduced over a number of analytical readings of the codes in order to permit sensible interpretation.

## 3. Results

**Figure 1:**
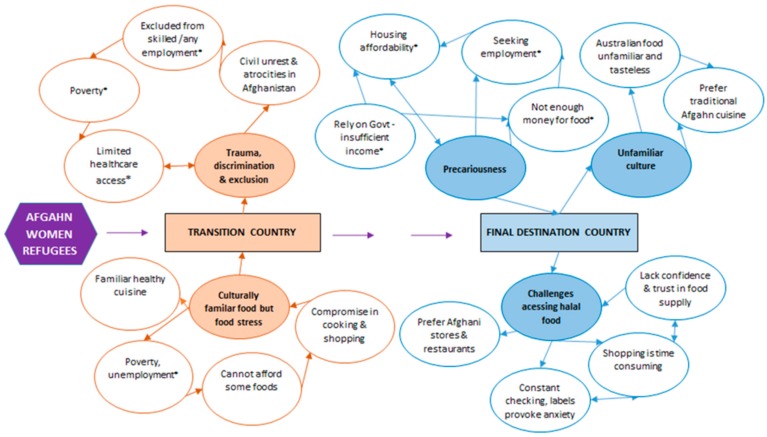
Themes and subthemes of Afghani women’s experiences of migration, food and stress during the migration journey from transition to final destination country. Key: solid orange circles = main themes in transition countries (Iran or Pakistan), white circles with orange borders = subthemes. solid blue circles = main themes in destination country (Australia), white circles with blue borders = subthemes. Uni-directional arrows are used to indicate a subtheme which has arisen from a main or parent theme. Bi-directional arrows are used to show an inter-relationship between subthemes. Sub-themes with an asterisk reflect a key social determinant of health which may influence individual or community health.

Ten women who had been living in Australia for 2 years or less were interviewed between May and September 2017. The stated length of time women had been living in Adelaide specifically ranged from 3 months to 2 years. Before coming to Australia, seven women had transitioned via Iran and two via Pakistan and one woman did not migrate via a transition country. Five key themes and associated subthemes emerged from the data as shown in Figure 1. In the transition country, these were (i) trauma, discrimination and exclusion and (ii) familiar food culture, but food stress. In the final destination country (Adelaide, Australia) these were (iii) precariousness, (iv) an unfamiliar food culture and (v) challenges and difficulties in accessing halal food.

### 3.1. Trauma, Discrimination and Exclusion (in the Transition Country)

The women interviewed had come to Australia from Afghanistan via transition countries such as Pakistan or Iran as part of their migration journey. The war in Afghanistan impacted on the safety as well as, social and economic capacity of interviewees and was the reason for emigration and to seek for better life opportunities. For example, some participants noted Kabul was too dangerous for work, or to engage in social or education activities.

P5: *We lived in rural area in Kabul and I was only able to do my education up to primary since I did not have any facilities with transportation to the school … Kabul was very unsafe place and always a war zone.*

In transition countries, interviewees reported living in poorer areas in close proximity with other refugees from their country to provide a sense of connection and safety, yet exposure to violence and trauma also occurred.

P7: *… Our community get attacked by Pakistani people. I remember when I was 13 and we lived in a rural area and there was a blast and many people from my community got killed …*

P4: *Yeah, we lived in one of the dangerous areas of Pakistan which has been targeted for 15 years by the Taliban. We had a blast next to our land and it killed more than a hundred people at time. We had seen a lot of body parts and everything just came into our house. It was that close. So that why we thought that, no, we cannot live here anymore now, and we cannot go back to Afghanistan as well because the situation there is even worse, so we decided to move to Australia.*

Discrimination on the basis of refugee status, gender, race or religion was reported by some interviewees in their transition country. One interviewee believed that discrimination occurred because of the perception that Afghani refugees took away employment opportunities from local Iranian or Pakistani citizens. Afghani refugees were not eligible to work in the transition country and working illegally carried significant risks. When work was available it was often unskilled labouring jobs.

P8: *The only employment was being a cheap labourer. Even with a university degree, employers were hesitant to employ us because of the high likelihood of being deported to Afghanistan if the rules changed.*

Access to education in transition countries was limited and if available was crowded, disproportionally expensive for refugees or required a specific documentation (visa or identity card), which they did not have.

P10: *Because we were Afghani, we had less right than local people and for example for getting admission for schools there was heaps of bureaucracy work between different department … there was a big discrimination for you as an Afghani refugee. It was really hard to have any hope for your future. In schools there was a lot of discrimination and they did not want us to progress.*

P10: *In Iran we studied in a public school for a while then they had a rule and removed us from public school, and we had to go to private schooling which was very expensive.*

Health care access in transition countries was limited, expensive i.e., they were often charged higher fees than locals, and health insurance was not an option.

P7: *We did not have access to health services in our place and we had to travel to the next town and they would charge us more than the locals and we could not afford that.*

### 3.2. Familiar Food Culture, but Food Stress (in the Transition Country)

The cultural similarities and familiarity of foods between the transition country (Iran) and Afghanistan was noted by several interviewees. Both countries had similar cuisines and religious traditions, for example the adherence to halal foods. The familiar food culture made it easier for some women and their families to find and purchase recognised ingredients and adapt to transition country life.

P3: *There was not much difference between Afghani and Iranian cuisine so my parents were able to adapt very easily.*

P8: *Food in Iran was healthy and had good quality. It was very similar to our own traditional foods.*

However, despite the alignment of food culture in the transition country, the experience of financial insecurity due to unemployment and the higher costs of living or studying had a flow-on effect to food. Staple food items such as meat, lentils and fruit were expensive and meal preparation became restricted and repetitive based on a limited variety of ingredients. The cost of red meat was out of reach or only small amounts were purchased monthly. Low income also imposed restrictions on access to transport with respect to food shopping.

P10: *Food in Iran was really good since it was a halal food but the only problem was its price which was not affordable for families like us. Sometimes my dad did not get his salary on-time and my mother had to make a lot of compromises to her cooking ingredients. We could not afford red meat specially most of the times.*

P4: *We didn’t eat meat that much because meat was too expensive for us [in Pakistan]. Even the people used to say that lentil is the cheapest food that anyone can afford, but even the price of lentil have become so high that it was above the pay of a person … So, we never could not afford it.*

Over time a compromised diet impacted on some interviewees’ health with deficiencies diagnosed as P3 explains “*whenever we go to a GP, we diagnosed with calcium and iron deficiencies*”.

### 3.3. Precariousness (in Australia)

Despite being safe and settled in Australia, and having access to government support for health, housing and education, some women and their families were struggling. A sense of precariousness was mainly experienced in terms of sufficient money, access to affordable, suitable and secure housing and gaining secure employment with limited English proficiency. Many respondents were keen to take up study opportunities in order to retrain with the view of achieving financial independence and owning their own home.

P10: *Finding an employment for us is also challenging considering we do not have local experiences and fluency in English.*

P4: *Still I don’t have a job. I am talking to the job seeker to try and get me a part time job, but because of my studies they are getting so much tougher and it is fulltime, so it was not possible and the job offering me was to do work in a pork factory, so we are not comfortable with it.*

P4: *I want to support my family. Now we are living in a rent house, so we need to buy house, that’s why I need to have a job for that.*

Without employment, some interviewees relied on extended family members or in-laws for additional support. Those on government welfare payments from Centrelink struggled with the cost of rental housing and other living expenses with government payment levels deemed inadequate for “living a good life”.

P2: *My husband and I could not survive on Centrelink money without the support of my father-in law and could not afford to rent a place to live.*

P4: *Over here most problems I have faced was driving. It was too difficult for me to get a driving license because we have to pay a lot for every license that we have to get. The driving test we must pay is too much, because I failed my first driving test, it was $250.*

Access to rental housing was also tenuous because of mistrust by landlords about their ability to meet the rental payments whilst living on government welfare

P1: *In Australia we have difficulty finding a place to rent for several reasons. First, we are a big family and second, our family relies on Centrelink payment and landlords are not trusting us that we can afford their rent.*

There were mixed views on how financial precariousness (resulting from being on government assistance payments), impacted on food. For some interviewees the high level of food affordability in Australia was noted compared to Iran, despite a reliance on government welfare payments.

P10: *In Iran if we can afford cheese or butter for our breakfast we felt that we eating like a rich people but in here we can afford to eat variety of things for example egg, jam, milk, walnut, honey all in one meal which is amazing.*

P6: *I live with my cousin and rely on government money to live. I can afford food shopping, but I cannot afford other shopping like clothes etc.*

Additionally, for others,

P1: *Now that we are not living in governmental house, the rent and bills is taking up most of money and we need to watch how we spend leftover money on food and we will buy food in bulk to save some money or we will only shop at cheap markets such as Sunday market.*

P7: *Support for refugees to find good quality cheap food is really important as they might pay for their house, bills and education and or as an elder son they are supporting the whole family, this will leave small budget for the food.*

Only one woman spoke of coping with episodes of running short of food—in Pakistan neighbours helped, but in Australia, she relied on friends.

P7: *In Pakistan if we run out of a food, we will ask our neighbours because neighbours there are in relationship but here in Australia this is not possible. Here I made a lot of friends and I will ask them if I need.*

### 3.4. An Unfamiliar Food Culture (in Australia)

Lack of familiarity with food in Australia was a common experience and was at odds with their experience of food in the transition country, which was halal, and hence familiar. In Australia, the limited availability of traditional foods or products, the taste of food and uncertainty about new foods or cuisines were often mentioned. In particular the food in Australia was consistently described as “tasteless”:

P5: *When I came to Australia two years ago, food was tasteless, and I even do not like the taste of water here but later on I got used to it.*

P2: *Chicken here is tasteless, and I don’t like that taste of dairy and egg either here and I would not eat them.*

Traditional Afghani cuisine was preferred over other types of food or cuisines available such as Italian or Japanese as they were seen as more desirable, tastier and more filling.

P10: *I have not tasted Australian food … but my sister once has tried Sushi and she could not eat it as its odour is like a raw fish which is not pleasant for us. I do not know why this food is so popular here.*

P9: *We eat traditional food here in Australia. I think Australian foods are healthier, but I would prefer our own food as it is tastier.*

Consequently, many women preferred to maintain a traditional diet in Australia and either ate at home or in Afghani restaurants. P7 summarised the position of several women succinctly:

P7: *I do not know what the Australian food means, and I will eat traditional food most of times …*

### 3.5. Challenges in Accessing Halal Food (in Australia)

Many interviewees were highly committed to strictly following the halal dietary component of their religion; however, this also created a degree of stress for them in finding appropriate foods locally.

P3: *One of my biggest concern over coming to Australia was that I brought up in a very religious Muslim family and I am very strict about halal food and I had a big stress that how I am going to maintain it here in Australia.*

When shopping in mainstream Australian supermarkets, many women expressed a lack of confidence and trust in being able to know which food products contained halal ingredients. Reading and understanding food labels was challenging with their limited English proficiency. Considerable effort and vigilance were required to “check everything” in order to ensure adherence to halal foods. This was a source of anxiety and was also time-consuming.

P4: *Even in the vegetables, they say ham flavour, so we have to check everything.*

For many women the only trusted source of familiar ingredients and halal foods were Afghani or Iranian specialty shops. These shops could be a considerable distance away from their home and transport was sometimes an issue.

P10: *We were lucky to live in a suburb (Prospect) close to Afghani shops and bakeries which have halal foods, but I have friends that they have to travel a long way from Salisbury or Elizabeth to come to shop at these places.*

P9: *In Australia we need to buy food that we can eat that is why we would only shop from Iranian or Afghani shops. We will eat out once a week only at Afghani restaurants.*

Most interviewees described a dualism in their food procurement, that is they shopped at trusted small Afghani stores for specific halal foods or traditional ingredients as well as at major supermarket chains to make savings. For some women, it was ‘risky’ to shop at supermarkets, so they only shopped there to buy household cleaning items, as P10 explains:

*I would not shop from Australian shops as they are less likely to have halal food and I only buy tissue or washing liquids* (*from supermarket*).

Other women had adapted and learnt to shop in major supermarket chains for halal foods. They did this by relying on friends, using a mobile phone App or looking for a specific code on the product label which indicated it was halal.

P10: *My friends from Afghanistan mainly and I have an App that you can upload your food and you can get ideas about which food you can make for your next meal.*

P3: *I always worry when I do not shop from Afghani supermarkets and I will check all the ingredients specially for gelatine and alcohol. I still cannot trust foods here that they are halal. I have heard that if they have a code it means that it is not halal.*

The lack of confidence and trust in accessing halal foods also extended to eating out in local restaurants other than Afghani or Iranian. This was a new phenomenon for some women and was different to their experience in Iran, where eating out was possible due to the similar food cultures which afforded them some “peace of mind”. Consequently, many women and their families only ate out in Afghani restaurants and there was a recognition that as they got busier with life in Australia, they tended to eat out (in Afghani restaurants) more often.

P7: *Whenever I go to a restaurant for example Chinese restaurant, I am not comfortable and if I ask about halal food, normally they have no idea and they will get really confused.*

P1: *We cannot eat in most restaurants as their food contains alcohol but in Iran, we had peace of mind when we shop or we eat outside.*

## 4. Discussion

The aim of this paper was to explore the migration journey and food experiences of Afghani women refugees in both their transition country (Iran or Pakistan) and in Australia. Five themes emerged from the interviews; (i) trauma, discrimination and exclusion, (ii) culturally familiar food, but food stress (in the transition country, Iran or Pakistan), (iii) precariousness, (iv) unfamiliar food culture and (v) challenges accessing halal food (in the final destination country, Australia). The findings indicate that in both the transition and destination countries, participants experienced challenges in meeting their food needs which resulted from a wide range of personal, social and environmental determinants that prevailed during their migration journey. Stress associated with accessing food was evident in both transition and destination countries and occurred primarily as a consequence of poverty and exclusion from the labour market.

### 4.1. The Transition Country Experience

In the transition country, the women and their families faced considerable poverty, discrimination and a sense of insecurity, factors that are recognised to have negative impact on both mental and physical health outcomes [19,42,43]. Discrimination in particular, is one of the stressors, for which when uncontrolled and unpredictable—similar to what these women would have experienced in transition countries—is significantly harmful to health outcomes, such as high blood pressure [44,45]. Discrimination as one of the social determinants of health, influences the socioeconomic opportunities for population groups as well as the quality of services they would access [46].

The experience of poverty was underpinned by being excluded from the ability to work professionally in the transition country as a condition of their refugee status. Some family members accepted precarious employment for example, unskilled jobs to earn cash, however they risked contravening the conditions on which refugee status was conferred and subsequent repercussions. Social exclusion such as being excluded from employment opportunities, access to health care and education was evident in the data and are key social determinants of health. Unemployment and precarious employment have a strong impact on physical and mental health and wellbeing such as poor quality of life, risk of occupational injuries, especially for vulnerable young people [47]. The SDoH recognises poor access to employment opportunities, low levels of education and poverty as significant determinants of health [43]. Access to affordable education and healthcare was also difficult due to poverty and discriminatory practices that required refugees to pay higher fees for services.

Despite the similarities in participants’ food cultures between the country of origin and in the transition country, financial poverty contributed to food insecurity and consequently poorer nutrition [48,49]. Various vitamin deficiencies are commonly found amongst refugees upon arrival in the final destination country [50]. A recent study of newly arrived refugees to Sydney, three quarters of whom were from the Middle East, found women had less anaemia than other refugees but higher levels of Vitamin D deficiency [51]. Living in poverty resulted in a degree of food insecurity, which occurs when there is discrepancy between the cost of healthy food relative to household income, but is not related to food access [52].

### 4.2. The Australian Experience

In Australia, women found foods were unfamiliar, and they experienced anxiety in finding halal foods. This is not surprising, as the cultural construction of food has been noted to influence migrants’ access to food in the final destination countries [24]. For new migrants and refugees, a combination of factors, including difficulty finding familiar foods and learning to eat unfamiliar foods, are recognised as important determinants that influence their health [19,53]. In spite of receiving some government welfare payment, a small number of Afghani women experienced financial pressures and concomitant food stress that had some similarities to their experiences in the transition country. In this respect, the experience of refugees is not dissimilar to other households reliant on welfare, who are known to experience food stress [52] and are more likely to experience food insecurity [54].

On arrival in Australia, Afghani women had difficulty in accessing familiar food, reported housing precariousness and many strived to achieve financial independence with the view to home ownership. This is not surprising given employment, housing and sense of belonging are important social determinants of health [43], and necessary in achieving a healthy settlement [55]. The lack of, or the perception of lack of these, could continue to be stressors in Afghani refugees’ lives in Australia, contributing to a vicious cycle of poor socioeconomic status and poor health outcomes for these migrants, their families and communities. These findings support other studies where similar themed patterns have emerged, with new migrants preferring to maintain their food practices and behaviours [17], but are challenged to do this given they are unfamiliar with not only the food, but other social factors and systems in their final destination country [55,56].

Adaptation to new food patterns by new migrants depends on their ethnic/cultural background, the foundation and strengths of their religion and ideologies and the extent to which they want/need to adapt food from the new environment [24,57]. The experiences of poverty, food stress and anxiety about accessing halal food in Australia reinforced a sense of precariousness in Afghani refugee women’s lives. The challenges in accessing halal foods in Adelaide was a key finding as it created some anxiety, as has been identified in a study on trust in food labelling in Australia [58]. Halal food refers to foods derived from animals slaughtered and prepared according to Islamic slaughter rituals [59] and is central to Muslim self-identity. A theoretical framework to explore the drivers of consumption of halal foods in non-Muslim majority countries has been proposed by Mumuni et al. (2018) [59]. They found that an insistence on religiously motivated behaviour is a strong predictor of consumers’ willingness to exert effort towards an insistence on halal food.

In the current study, concerns about the authenticity of halal foods available in Adelaide supermarkets resulted in many participants limiting their food procurement to mainly small Afghani specialist shops. This is consistent with other studies in non-Muslim majority countries such as the UK and Belgium [60,61] and a study in Australia [58]. A UK study found that whilst the majority of UK Muslims considered halal meat sold from major supermarkets was more hygienic and better quality than meat from local butchers, they were unlikely to shop at supermarkets due to a lack of trust in the authenticity of the halal products. Given the evidence from similar studies elsewhere [58], it is reasonable to hypothesise that improving trust in the authenticity of halal food would have an influence in minimising the complexities of food access in the current study participants.

Notably in our study, a few women utilised a mobile phone app to check the authenticity of supermarket halal foods. The halal food market is a rapidly growing global business and Australian smart phone apps such as *Halal Advisor* are assisting Muslim consumers to verify halal food in supermarkets and locate appropriate food in cafes and restaurants [62,63]. However, this was not the case for all Afghani women. Additionally, although Australia provides opportunity to eat out with families in a wide range of eateries, for Afghani women, this was limited to Afghani restaurants, where they trusted the food would be halal, reiterating that food is socioculturally and religiously constructed [24,59]. While public health approaches encourage the retention of traditional diets to minimise consumption of energy dense highly processed foods, in order to continue the ‘healthy migrants effects’, it is also advantageous for migrants to adapt to local foods in order to achieve a healthy settlement [19,53].

### 4.3. Study Strengths and Limitations

This paper is the first Australian work reporting specifically on the food experiences of Afghani women, both in their transition country as well as the destination. The major strength of this study is that it provided a comprehensive synopsis of the various social determinants of health affecting food and life experiences of this cohort in the transition country and in Australia.

Scant research investigating the food experiences of refugees arriving in Australia exists, and as such these findings are limited to the experiences of Afghani women living in Adelaide who participated in the study. Only 10 women were interviewed, and this does limit the extent to which we can apply findings more broadly. However, as noted saturation was achieved and is the usual criteria in defence of small numbers of participants. Closely aligned with this was the process used to generate themes. This was done from the beginning of the interview process so that emerging themes could be checked out with subsequent participants [64] thus confirming the point of saturation.

The participants in this study were young, recently arrived women; some had permanent humanitarian visa’s and the rest were sponsored by their spouse. This has probably influenced both the level of government support received, as well as the level of resident family support and consequently their life and food experiences in Australia. Refugees arriving on temporary visas will have different experiences due to the different visa specific entitlements. Our cohort represents a specific group of refugees which is homogenous. However, their experiences do not represent all Afghan refugees including males, those of different ages and visa types, as well as individuals with different lengths of stay. Nevertheless, the overall internal validity of this study appears to be strong which suggests the results are reliable and therefore of practical use for understanding and developing appropriate supports for dietary acculturation in a new country.

Further research could focus on (i) a larger, more demographically diverse (e.g., age, gender, education level and length of time in Australia), sample of Afghan migrant women to see if the findings change greatly, and (ii) the food experiences of the Afghan refugees who settle in other countries, where cultural difference may be smaller.

A methodological strength of this work was the use of a native Farsi speaker. This was crucial to foster engagement by building trust, rapport and seeking the endorsement of local community leaders, promoting recruitment and conducting culturally appropriate interviews and eliciting quality data. Further research validating the lived food experiences of Afghani women, their coping strategies and investigating pathways out of poverty are warranted. Further research is needed to identify further ways to support people to engage and trust the local food system.

## 5. Conclusions

This study highlighted the ways in which Afghani women described the stress associated with food access and provisioning during the migration and settlement challenges. The ability of Afghani migrants to maintain their cultural foods and for a healthy settlement of their families was affected by structural factors such as poverty and poor employment opportunities in Australia. Structural and physical barriers to accessing cultural foods existed, notwithstanding relief provided by specialty shops and restaurants around Adelaide established by earlier settlers from Afghanistan. More needs to be done to improve the compliance of the food supply to halal requirements, so that consumer information to new immigrants is easily accessible and trustworthy. As the numbers of Afghani refugees is increasing in Australia, it is necessary to address social determinants by increasing pathways to employment and finance acquisition opportunities to alleviate poverty. The research demonstrates that while food stress was experienced in both the transition and country of destination, and in both cases was the result of poverty, there are variations in how the social determinants of food stress play out. In transition countries poverty is aligned with racism and lack of access to services, while in Australia while access to social services is assured, there are still difficulties in accessing appropriate food and problems with language, housing and employment. The first has its origins in racism, the second in significant cultural and religious differences. Different structural, cultural and political factors come into play across both countries requiring the women to learn and re-learn how to manage the provision of food. This suggests that policy makers and health advocates need to understand the nuances, not just of food choices, but those determinants of health peculiar to the migrant or refugee’s situations.
