# Addendum: Kavian, F.; et al. Migration, Stress and the Challenges of Accessing Food: An Exploratory Study of the Experience of Recent Afghan Women Refugees in Adelaide, Australia. *Int. J. Environ. Res. Public Health* 2020, *17*, 1379; doi:10.3390/ijerph17041379

**DOI:** 10.3390/ijerph17134808

**Published:** 2020-07-03

**Authors:** Foorough Kavian, Kaye Mehta, Eileen Willis, Lilian Mwanri, Paul Ward, Sue Booth

**Affiliations:** 1 College of Nursing and Health Sciences, Flinders University, GPO Box, Adelaide 2100, Australia; Foorough.Kavian@flinders.edu.au (F.K.); Kaye.Mehta@flinders.edu.au (K.M.); Eileen.Willis@flinders.edu.au (E.W.); 2 College of Medicine & Public Health, Flinders University, GPO Box, Adelaide 2100, Australia; Lillian.Mwanri@flinders.edu.au (L.M.); Paul.Ward@flinders.edu.au (P.W.)

In the original version of our article [1], insufficient details were given in recognition of the support and funding from our Canadian collaborators. We apologize for the original error. To correct this oversight, the acknowledgements have been altered in the “Funding” section to more appropriately acknowledge the contribution of Hassan Vatanparast and Mahasti Khakpour, who conceptualised the grant and broader research studies, both quantitative and qualitative.
